# Correlation Between Benign Paroxysmal Positional Vertigo and 25-hydroxyvitamin D

**DOI:** 10.3389/fneur.2020.00576

**Published:** 2020-06-23

**Authors:** Penglong Song, Xianshu Zhao, Yanjun Xu, Zhigang Zhao, Li Wang, Yang Liu, Qian Gao

**Affiliations:** ^1^Department of Otolaryngology/Head and Neck Surgery, The First Affiliated Hospital, Harbin Medical University, Harbin, China; ^2^Health Center of Screening and Prevention of Diseases, The First Affiliated Hospital, Harbin Medical University, Harbin, China; ^3^Department of Otolaryngology, Harbin Second Hospital, Harbin, China

**Keywords:** benign paroxysmal positional vertigo, 25-hydroxyvitamin D, otoconia, estrogen, lower, vitamin D

## Abstract

**Objective:** The correlation between benign paroxysmal positional vertigo (BPPV) and vitamin D levels was controversial. We explored age- and sex-related effects on 25-hydroxyvitamin D (25(OH)D) and correlation between 25(OH)D levels and BPPV.

**Subjects and Methods:** We recruited 380 patients with BPPV and collected 25(OH)D records of 3,125 control subjects who were further divided into age- and sex-based subgroups. We respectively investigated the effects of sex and age on 25(OH)D by comparing sex- or age-based subgroups. Then, we separately compared levels of 25(OH)D in sex-and age-based subgroups between the BPPV and control group.

**Results:** 25(OH)D levels in male subgroups were significant higher than those in female subgroup both in the BPPV and control group. With increasing age, 25(OH)D levels gradually increased, and there were significant between-subgroup differences for age in the control group. In males, the significant between-subgroup difference was observed only in the <40 year subgroup. Three female age-matched subgroups (<40, 40–49, and 60–69) showed significant between-subgroup differences.

**Conclusions:** There are sex and age differences in vitamin D levels. For both male and female patients with BPPV aged <40 years and female patients with BPPV aged 40–49 and 60–69 years, the lower vitamin D level is a risk factor for BPPV. In female patients with BPPV aged 50–59 and >70 years, and male patients with BPPV aged >40 years, the correlation between vitamin D and BPPV is non-existent.

## Introduction

Benign paroxysmal positional vertigo (BPPV) is the most common type of peripheral vestibular vertigo, with a lifetime prevalence of 2.4% and believed to be an otoconia-related balance disorder (1, 2). The pathophysiological processes of BPPV are well-established and involve falling into semicircular (canalolithiasis) or attaching to the cupula (cupulolithiasis) of otoconia debris which change the sensitivity of semicircular canals to gravity. Known predisposing factors for BPPV include advanced age, head trauma, vestibular neuritis, Meniere's disease, migraines, otologic surgery, and prolonged bed rest (3). However, there is little data on the underlying causes of otoconia degeneration and otoconial membrane detachments.

Vitamin D deficiency is a significant public health problem worldwide that affects almost all age groups. Approximately one billion people are affected by low vitamin D levels (4, 5). Besides modulating bone homeostasis, the use of vitamin D to prevent and treat non-skeletal health issues has gradually received significant media and research attention in recent years. In humans, observational data has suggested a link between poor vitamin D status and a large number of major human diseases including cancer, muscle weakness, falls, infections, autoimmune diseases, hypertension, cardiovascular disease, obesity, diabetes, metabolic syndrome, and other health problems (6, 7).

Previous studies have shown a link between seasonality variation, serum level of vitamin D and BPPV (8–13). However, some dissenting scholars believed that the correlation of vitamin D levels with BPPV cannot be proven by existing data and the observed coexistence of BPPV with vitamin D deficiency is coincidental (14, 15). Whether vitamin D levels is related to BPPV is still controversial. Furthermore, based on literatures quoted above, we found that almost all researchers did not seriously consider the possible effects of sex and age ratio differences (effect of sex and age differences on vitamin D status) on the results in their studies on the correlation between vitamin D and BPPV. In this study, we clarified if there were sex and age differences in vitamin D levels and then explored the correlation between vitamin D levels and BPPV after entirely eliminating the effect of sex and age on vitamin D levels by grouping.

## Materials and Methods

We identified 380 consecutive patients with first diagnosis of idiopathic BPPV from the First Affiliated Hospital of Harbin Medical University dizziness clinic between September 2015 and November 2018. These individuals included 283 females (age range = 19–85 years, mean age ± SD = 50.5 ± 13.5 years) and 97 males (age range = 21–83 years, mean age ± SD =51.3 ± 13.8 years). Diagnoses were confirmed via medical history and positive provocative maneuver (either Dix-Hallpike or Roll test). Among the 380 patients, 268 were diagnosed with posterior semicircular canal canalolithias, 69 were diagnosed with horizontal semicircular canal canalolithias and 43 were diagnosed with horizontal semicircular canalcupulolithiasis. In addition, we collected 25(OH)D records of 3,125 control subjects, including 1,919 females (age range = 18–92 years, mean age ± SD =50.6 ± 15.2 years) and 1,206 males (age range = 18–96 years, mean age ± SD =51.7 ± 15.1 years) from the Screening and Prevention of Disease health center of the First Affiliated Hospital of Harbin Medical University between January 2017 and December 2017. Fasting early morning venous blood from both BPPV patients and control subjects were measured serum 25-hydroxyvitamin D (25(OH)D) levels using the automatic chemiluminescence immunoassay analyzer (Liaison XL, Type 2210, DiaSorin S.p.A, USA) and concentrations between 30 and100 ng/ml were considered normal. The study excluded patients with comorbidities including Meniere's disease, vestibular neuritis, head trauma within three months, vestibular migraine, those with a history of total thyroidectomy, patients who took calcium or vitamin D therapy for one year before the study, or those with histories of prolonged bedrest secondary to orthopedic surgery within the past six months. The Ethics Committee of the First Affiliated Hospital of Harbin Medical University approved the study. All patients who could be personally contacted gave consent for publication. Collected information was anonymized by code numbers and solely used for this study.

This study was divided into two parts. In the first part of this study we clarified whether age and sex had an effect on vitamin D levels. First, both the BPPV and control group were each divided into two subgroups according to sex (male and female). By comparing 25(OH)D level in male subgroup with that in female subgroup, we investigated the effects of sex on vitamin D levels in the BPPV and control group, respectively. Then we investigated the effects of age on vitamin D levels, which was divided into two steps. The first step, the linear correlation between age and vitamin D was tested by linear by linear association in the control group. The second step, the control group was divided into subgroups according to age (18-29, 30-39, 40–49, 50–59, 60–69, 70-79, and >80 years). Six control subjects aged over 90 years were classified into >80 year subgroup. We investigated the effects of age on vitamin D levels by comparing 25(OH)D levels between different age-based subgroups.

In the second part of this study, both the BPPV and control groups were each divided into subgroups according to sex (male and female) and age (<40, 40–49, 50–59, 60–69, and >70 years). The BPPV patients in both 18–29 and 30–39 age segment were sparse in this study and there was no statistical difference in vitamin D levels between 18–29 and 30–39 year subgroup in control group which had been validated in the first part of the study, hence we classified the BPPV patients under 40 years old into <40 age subgroup. Likewise, we classified the BPPV patients over 70 years old into >70 age subgroup. We matched the two subgroups with the same sex and age segment from the BPPV and control group respectively. By comparing 25(OH)D levels of the two matched subgroups, we separately investigated the correlations between vitamin D and BPPV in each sex and age segment.

### Statistical Analyses

Statistical analyses were performed using IBM SPSS Statistics 19 for Windows. The correlation between age and 25(OH)D levels was tested by linear-by-linear association, one-way ANOVA and followed multiple comparison tests. In multiple comparison tests, Tamhane's T2 is appropriate when the variances are unequal. Between-subgroup comparisons were made by using *T* test, ANOVA test and, if necessary, Welch's ANOVA. The Mann-Whitney test was also used when the sample size of any subgroup was <30. A value of *P* < 0.05 was considered statistically significant.

## Results

**Figure 1:**
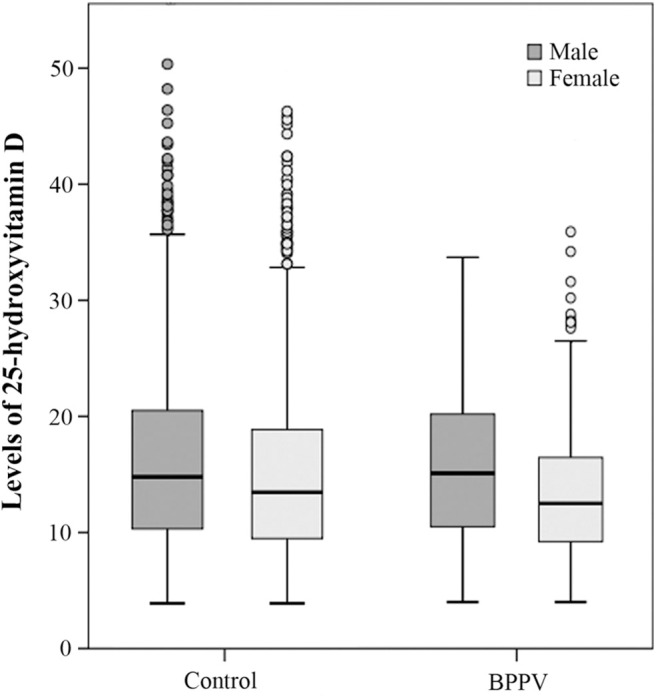
Box blots of 25-hydroxyvitamin D levels in females and males in both the control and BPPV groups. The dark gray box blots indicate male levels of 25(OH)D and the light gray box blots indicate female levels of 25(OH)D. Each box indicates a percentile range of 25–75%, the lines through the boxes represent the medians, and the range lines indicate the upper and lower values that are not classified as statistical outliers. Circles denote 25(OH)D concentrations that are statistical outliers.

In the control group, the 25(OH)D level in male subgroup was higher than that in female subgroup and the sex-based difference was statistically significant. (T = 4.086, *P*_control_ < 0.01). In the BPPV group, the male 25(OH)D level was also higher than the female 25(OH)D level (Figure 1). The sex-based difference was also statistically significant. (T = 2.996, *P*_BPPV_ < 0.01) These results confirmed that there were sex differences in vitamin D levels which were higher in males than in females.

**Figure 2:**
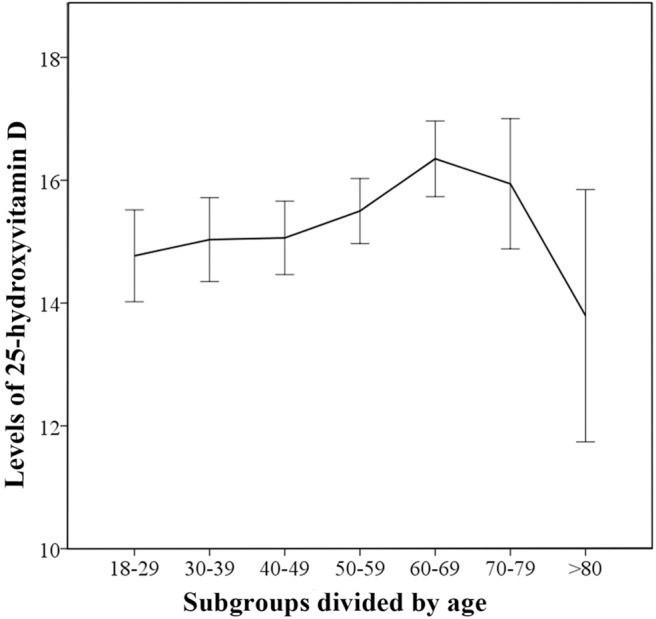
Trends for 25-hydroxyvitamin D levels in different age-based subgroups for control group. The median 25(OH)D levels in the age-based subgroups are indicated and error bars show 95% confidence interval.

In the control group, the 25(OH)D levels gradually increased with age, peaking for subjects in 60–69 subgroup (Figure 2). The linear-by-linear association showed that there was a linear trend between age and 25(OH)D levels. (Z = 8.192, *P* < 0.01) There were significant between-subgroup differences for age in the control group by one-way ANOVA (F_welch_ = 2.954 *P* < 0.01) and the followed multiple comparison tests showed statistically significant differences in vitamin D levels between 60–69 and 18–29 subgroup. (*P*_Tamhane_ < 0.05) The results confirmed that there were age differences in vitamin D levels.

**Figure 3:**
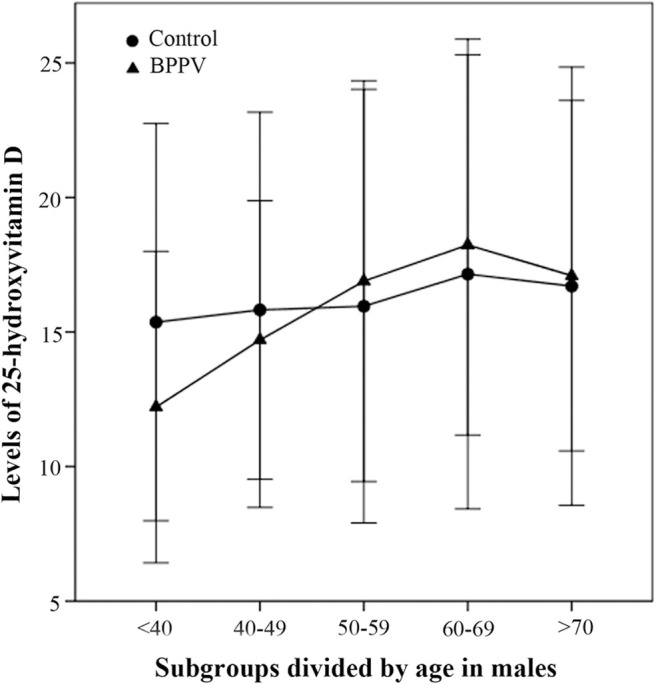
Comparison of male 25-hydroxyvitamin D levels between the BPPV and control subgroups divided by age. The median levels of 25(OH)D levels in subgroups divided by age are indicated by black circles in the control group and by black triangles in the BPPV group. Error bars show standard deviation.

In the second part of this study, the 25(OH)D levels of males in the BPPV group increased rapidly with age and peaked at age 60–69 years (Figure 3). The 25(OH)D levels of males aged <40 and 40–49 years in the BPPV group were, respectively, lower than those in the control group; however, the significant between-subgroup difference was observed only in the <40 year subgroup (*P*_Mann−Whitney_ <0.01). There were no significant between-subgroup differences for the other four male age-matched subgroups (40–49, 50–59, 60–69, and >70 year subgroups).

**Figure 4:**
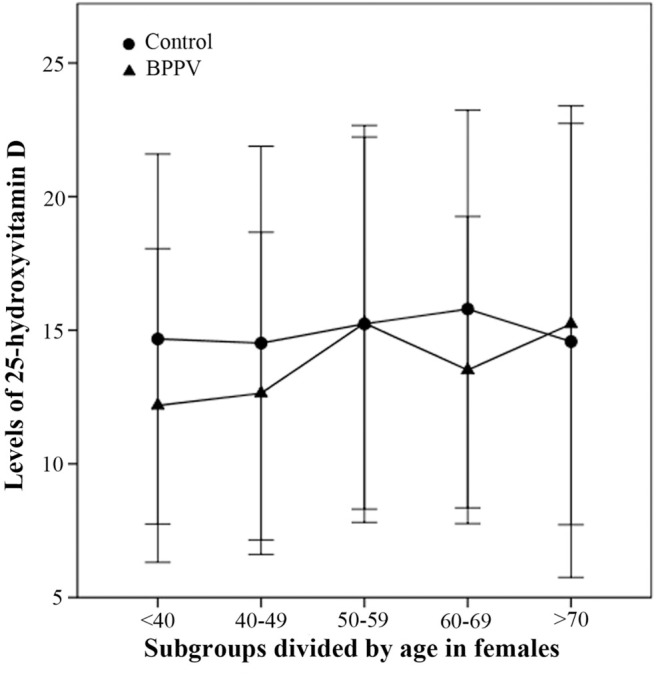
Comparison of female 25-hydroxyvitamin D levels between BPPV and control subgroups divided by age. The median levels of female 25(OH)D in subgroups divided by age are indicated by black circles in control group and by black triangles in BPPV group. Error bars show standard deviation.

In females, the 25(OH)D levels of females aged <40, 40–49, and 60–69 in BPPV group were respectively lower than those in the control group (Figure 4). Three female age-matched subgroups (<40, 40–49, and 60–69) showed significant between-subgroup differences (T = 2.673, *P* = 0.008 for <40; T = 2.281, *P* = 0.024 for 40–49; T = 2.524, *P* = 0.014 for 60–69). There were no significant between-subgroup differences for the other two female age-matched subgroups (50–59 and >70) (Figure 4). The results of the second part in this study showed that correlation between vitamin D and BPPV was diverse and varied according to sex and age segment.

## Discussion

In the first part of the study, the 25(OH)D levels of males were significant higher than those of females both in the BPPV and control groups. So we concluded that sex can affect vitamin D status, which was consistent with some current studies (16, 17). In addition, we found that 25(OH)D levels gradually increased with aging which showed significant age-based differences. We believed that there were age differences in vitamin D levels which was also consistent with previous research results (18, 19).

On account of sex and age differences in vitamin D levels, in the second part of the study both the BPPV and the control group were each divided into subgroups according to sex and age segment, and the possible effect originated from sex or age ratio inconsistency can be entirely eliminated when comparing the vitamin D levels between the BPPV and the control group.

We found that the 25(OH)D levels of both males and females aged <40 years in the BPPV group were significantly lower than those in the control group. The findings confirm the close correlation between BPPV and 25(OH)D in BPPV patients younger than 40 years of age. We think that the lower vitamin D level is a risk factor for BPPV in patients aged <40 years.

The etiology of BPPV is widely believed that otoconia (calcium carbonate crystals) dislodge from the macula of the utricular otolith and enter the semicircular canals and/or ampulla of the semicircular canals (canalithiasis and/or cupulolithiasis, respectively). Otoconia results from ordered deposition of inorganic calcium carbonate crystallites onto a preformed framework, consisting of an organic matrix. The ultrastructure and function of the otoconial matrix for regulating crystal growth resembles that of bone turnover. Many studies confirmed that otoconia including its frame and organic matrix were in a process of constant renewal. The morphology of the otoconia of the rat utricle and saccule changed and the calcium content of the otoconia decreased after 160-days of tail suspension (20). Vibert et al. reported ultrastructural modifications of the otoconia in terms of changes in their aspect, size and density in ovariectomized osteoporotic female adult rats; the otoconia were increased in size and decreased in their density as compared to a control group of rats (21).

As turnover in otoconia is an ongoing process, the lower vitamin D level may disturb formation of otoconia or otoconial membrane by giving rise to disequilibrium of calcium homeostasis in vestibular endolymph, which may lead to the emergence of dislodged otoconia. The dislodged and undissolved otoconia ultimately induce endolymph flow on head movement or convert structures sensitive to angular acceleration into linear acceleration, which is the fundamental pathophysiological process in BPPV. Cao et al. confirmed in patients with BPPV that the particulate matter in the semicircular canals consisted of broken-off fragments of the utricular otolithic membrane with attached or detached otoconia (22).

It is important to maintain a low Ca^2+^ concentration in the vestibule endolymph because it prevent the production of abnormal otoconia which can result in dysfunction (8, 23). Moreover, an increased Ca^2+^ concentration in the vestibular endolymph can induce reduction in its capacity to dissolve the detached otoconia (21, 24, 25). It is known to all that the epithelial Ca^2+^ channel transport system, Na^+^/Ca^2+^ exchangers, and plasma membrane Ca^2+^ pumps expressed in the inner ear contribute to this low calcium levels in vestibular endolymph by transepithelial absorption of Ca^2+^. Yamauchi et al. confirmed epithelial Ca^2+^ channel transport system in semicircular canal duct could maintain low Ca^2+^ concentration in vestibular endolymph; particularly, they found that epithelial Ca^2+^ channel can be upregulated by 1, 25-dihydroxyvitamin D (26). Therefore, we speculate that the lower vitamin D level is likely to affect the formation of otoconia or resorption of detached otoconia by disturbing the calcium concentration of utricular endolymph in BPPV. This can also explain the findings that low vitamin D levels appear associated with recurrent BPPV and BPPV recurrences can be relieved with vitamin D supplementation (8, 9, 11). In recurrent BPPV patients, it is likely that the detached otoconia cannot be reabsorbed normally and eventually fall back into the semicircular canal again.

In female BPPV patients, 25(OH)D levels in the 40–49 and 60–69 age subgroups were both significantly lower than those in the corresponding control subgroups. Therefore, we think that the lower vitamin D level is also a risk factor for BPPV in female patients aged 40–49 and 60–69 years.

In this study, the females BPPV patients aged 50–59 years has the highest proportion in all BPPV patients (Table 1), which is consistent with the findings of other scholars (2). However, it is very special that the 25(OH)D level in 50–59 year subgroup was very close to that in the corresponding control subgroup, which showed no statistical difference. This implies that the vitamin D level have nothing to do with BPPV in female patients aged 50–59 years.

**Table 1:** 25(OH)D levels (ng/ml) and number in subgroups divided by sex and age.

	**Male**		**Female**	
	**BPPV**	**Control**	**BPPV**	**Control**
<40	12.21 ± 5.79 (17)	15.37 ± 7.38 (270)	12.18 ± 5.87 (60)	14.67 ± 6.92 (491)
40–49	14.70 ± 5.18 (21)	15.82 ± 7.34 (244)	12.64 ± 6.03 (70)	14.51 ± 7.37 (342)
50–59	16.89 ± 7.45 (27)	15.95 ± 8.06 (293)	15.27 ± 6.96 (79)	15.24 ± 7.43 (512)
60–69	18.24 ± 7.07 (25)	17.15 ± 8.73 (267)	13.51 ± 5.75 (49)	15.79 ± 7.44 (384)
>70	17.09 ± 6.51 (7)	16.71 ± 8.14 (132)	15.23 ± 7.51 (25)	14.58 ± 8.82 (190)

*25-hydroxyvitamin D levels are shown average ± standard deviation. The number in parentheses indicates the number of samples*.

Females usually start amenorrhea around age 50 when estrogen levels begin to rapidly decrease. The decline of estrogen weakens its inhibitory effect of on osteoclasts and therefore the activity of osteoclasts is increased. In addition, the rapid decline of estrogen also inhibits intestinal calcium absorption and reabsorption of urinary calcium, which in turn disturbs and causes loss of bone mass. As turnover in the otoconia is ongoing, such disturbances of calcium metabolism may generate failures in the remodeling of the internal structure and the attachment of otoconia on the otoconial membrane (25). A previous morphometric analysis revealed that the otoconia in ovariectomized rats shown larger volume and less dense than those in the control group (21). Yang et al. also revealed that estradiol deficiency was an essential risk factor for idiopathic BPPV in postmenopausal females (27).

It seems that estrogen deficiency, not the lower vitamin D level, is related to BPPV and lead to the highest BPPV incidence in female BPPV patients aged 50–59 years. Is it true? If estrogen deficiency is a risk factor for BPPV, females aged 60–69 years in this study should have a higher proportion than those aged 50–59 years because of lower estrogen levels along with aging. But the fact was that females aged 50–59 years had the highest proportion in all BPPV patients. In addition, it is particularly in this study that the female BPPV patients aged 60–69 years with the lower estrogen than those aged 50–59 years, showed statistically significant decrease in the 25(OH)D level compared with the corresponding control subgroup, which affirm the lower 25(OH)D level is a risk factor for female BPPV patients aged 60–69 years.

Estrone, a weak estrogen, is gradually become the primary ingredient of estrogen after amenorrhea in females. The estrone conversion rate in postmenopausal females is twice as fast as that in females of childbearing age and blood estrone concentrations range from 90 to 150 pmol/L. In postmenopausal women, although the estrogen levels are greatly reduced, the estrone which replaced estradiol can maintain basically normal physiological functions such as metabolic equilibrium of calcium. Thus, we conjecture that the lower vitamin D level and the rapid decline in estrogen are both risk factors for BPPV in female patients, and the correlation between vitamin D and BPPV in female BPPV patients aged 50–59 years is covered up by the rapid decline in estrogen caused by amenorrhea. Without the impact of sudden estrogen decline in females aged 60–69 years, we can detect it again that the lower 25(OH)D level is a risk factor for BPPV. It is the rapid decline in estrogen, not estrogen deficiency that led to the highest incidence of BPPV in females aged 50–59 years (28). It is well known that estrogen replacement therapy is beneficial to rehabilitate disordered calcium metabolism and prevent osteoporosis for females in perimenopausal period. We think that such strategies should be considered by doctors and involved in the treatment and prevention of BPPV in females undergoing perimenopause, which is consistent with the view of other scholars (29).

There were no significant between-subgroup differences in 25(OH)D levels for three male age-matched subgroups (40–49, 50–59, and 60–69 year subgroups). We think that vitamin D has nothing to do with BPPV in males aged 40–69 years.

For both male and female BPPV patients aged >70 years, the 25(OH)D levels were not lower but slightly higher than those in the control subgroup, which showed no statistical significance. We think that vitamin D levels has nothing to do with BPPV patients over 70 years old. Senile osteoporosis, formerly known as primary osteoporosis type II, has a particular pathophysiology. It appears very late in life, typically after 70 years old, and involves thinning of both the trabecular and cortical bones. On account of otoconia form in a manner similar to bone and primary osteoporosis type II also generally occur over 70 years old, we speculate that the occurrence of BPPV in adults older than 70 years is related to senile osteoporosis, which requires further research.

One limitation of this study is that in the BPPV group, the sample size in each male age-based subgroup was relatively small, which might compromise the generality of the results. The ratio of female to male for BPPV patients in this study was close to 3:1 and therefore the incidence of BPPV in males was obviously lower than that in females. Besides, the BPPV group was further divided into subgroups according to sex and age segment, which eventually led to small sample size in male age-based subgroups. Future research would expand the sample size, especially the male BPPV patients to consolidate the generality of the results. This study found the degree of correlation between the lower vitamin D level and BPPV in males was much lower than that in females. It was due to relatively small sample size in male subgroups or other reasons, which need further clarification in future research.

## Conclusion

We affirm that there are sex and age differences in vitamin D levels. For both male and female patients with BPPV aged <40 years and female patients with BPPV aged 40–49 and 60–69 years, the lower vitamin D level is a risk factor for BPPV. In female patients with BPPV aged 50–59 and >70 years, and male patients with BPPV aged >40 years, the correlation between vitamin D and BPPV is non-existent.

## Data Availability Statement

The raw data supporting the conclusions of this article will be made available by the authors, without undue reservation.

## Ethics Statement

The studies involving human participants were reviewed and approved by The Ethics Committee of the First Affiliated Hospital of Harbin Medical University. The patients/participants provided their written informed consent to participate in this study.

## Author Contributions

PS and XZ: design or conceptualization of the study, analysis or interpretation of the data, and drafting or revising the manuscript for intellectual content. YX: analysis or interpretation of the data, drafting or revising the manuscript for intellectual content. ZZ and YL: drafting or revising the manuscript for intellectual content. LW: revising the manuscript for intellectual content. QG: interpretation of the data, revising the manuscript for intellectual content. All authors contributed to the article and approved the submitted version.

## Conflict of Interest

The authors declare that the research was conducted in the absence of any commercial or financial relationships that could be construed as a potential conflict of interest.
